# Divergent morphological and acoustic traits in sympatric communities of Asian barbets

**DOI:** 10.1098/rsos.160117

**Published:** 2016-08-10

**Authors:** Anand Krishnan, Krishnapriya Tamma

**Affiliations:** 1 Department of Psychological and Brain Sciences, Johns Hopkins University, Baltimore, MD 21218, USA; 2 National Centre for Biological Sciences, Tata Institute of Fundamental Research, GKVK Campus, Bangalore 560065, Karnataka, India

**Keywords:** barbets, acoustic signal, niche divergence, morphological traits, community assembly

## Abstract

The opposing effects of environmental filtering and competitive interactions may influence community assembly and coexistence of related species. Competition, both in the domain of ecological resources, and in the sensory domain (for example, acoustic interference) may also result in sympatric species evolving divergent traits and niches. Delineating these scenarios within communities requires understanding trait distributions and phylogenetic structure within the community, as well as patterns of trait evolution. We report that sympatric assemblages of Asian barbets (frugivorous canopy birds) consist of a random phylogenetic sample of species, but are divergent in both morphological and acoustic traits. Additionally, we find that morphology is more divergent than expected under Brownian evolution, whereas vocal frequency evolution is close to the pattern expected under Brownian motion (i.e. a random walk). Together, these patterns are consistent with a role for competition or competitive exclusion in driving community assembly. Phylogenetic patterns of morphological divergence between related species suggest that these traits are key in species coexistence. Because vocal frequency and size are correlated in barbets, we therefore hypothesize that frequency differences between sympatric barbets are a by-product of their divergent morphologies.

## 1. Introduction

The patterns and processes influencing species coexistence in sympatric communities have long been a central theme of ecology research. Particular interest has focused on how the traits of species enable them to coexist, and on evolutionary patterns in these traits, which in turn influence community composition [1–3]. Community phylogeny is an emerging branch of ecology that seeks to understand these phenomena by investigating the phylogenetic structure of communities [4]. For example, environmental filtering results in similarity (clustering) of traits between co-occurring species, as an adaptation to a shared environment [3,5]. On the other hand, competition (in addition to competitive exclusion or ecological speciation) exerts the opposite influence on community composition, by limiting the coexistence of closely related species, resulting in trait divergence between co-occurring species [3,6]. If trait evolution were conserved, then we would predict that in a competition-driven scenario, communities are composed of a non-random sample of distantly related species (overdispersion), resulting in trait divergence. Alternatively, if traits show phylogenetic signatures of divergence between related species, then competitive interactions may establish communities either of related species, or a random sample of species from across the phylogeny (predictions reviewed in [3]). Thus, an understanding of trait structure within communities, as well as their phylogenetic structure and the patterns of trait evolution is required in order to delineate these two mechanisms of community assembly (i.e. environmental filtering versus competition) [2,7].

The morphology and behavioural traits of species influence various aspects of their ecology, and may thus influence species coexistence. Morphological traits are an important determinant of life history [8], and sympatric species may evolve divergent morphologies to minimize competition (ecological character displacement) by, for instance, specializing on different food resources [9,10]. Competition may also influence the evolution of sensory signals, e.g. acoustic signals, which serve a broad range of functions, such as territorial defence and advertisement to attract mates [11,12]. Divergent signals may additionally reinforce pre-mating reproductive isolation [13–17]. Thus, signal evolution may be influenced by adaptive mechanisms (such as environment and species ecology), sexual selection by mate choice, as well as neutral drift (reviewed in [11]). Regardless of which taxon-specific factors drive signal evolution, sympatric signallers (particularly close relatives) compete for acoustic space [18], and signals with similar temporal patterns and frequencies tend to mask each other [19–21]. The acoustic signals of related species may thus diverge temporally and/or spectrally (e.g. in frequency) to minimize competitive interference [22–24]. Studies in diverse animals such as crickets, frogs, bats and birds have found evidence for partitioning of acoustic signal space [25–32]. In passerine bird communities, sympatric species pairs are more divergent than allopatric pairs [33], and species living in more complex communities exhibit greater song stereotypy to avoid overlap [34,35], resulting in partitioning of acoustic space [36,37]. Other studies of passerine communities, however, have failed to find evidence of acoustic partitioning [38,39] suggesting signal convergence owing to shared habitats; in some cases, divergence may occur at the level of receiver adaptations [40,41].

Sympatric species with divergent acoustic frequencies also exhibit divergent morphologies [25,33,42], following a general pattern where acoustic frequencies and body size are negatively correlated [43,44]. This size–frequency relationship, together with the absence of partitioning in several passerine communities, raises the question: is acoustic frequency divergence in bird communities a result of adaptive processes to minimize masking interference, or merely a consequence of divergent morphological traits (i.e. ecological niche divergence)? In order to distinguish these scenarios, we must first understand the phylogenetic structure of communities, and phylogenetic signal in both morphology and vocal frequency. In a competition-driven scenario (or competitive exclusion, see above), we would predict that communities should show both phylogenetic overdispersion and trait divergence. We may then investigate putative mechanisms establishing these communities by understanding patterns of trait evolution (or phylogenetic signal, as in [3]).

**Figure 1.**
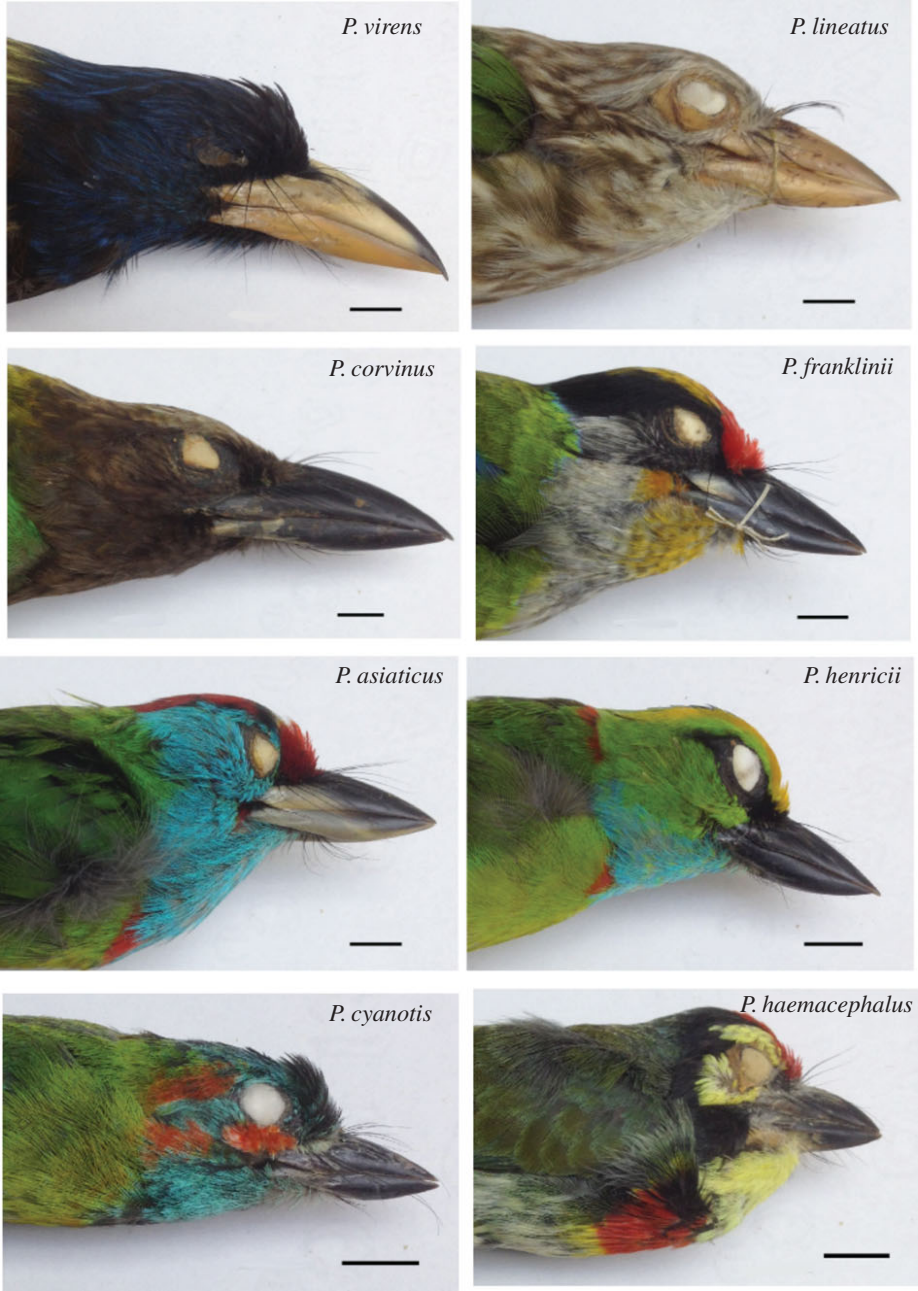
Diversity of size and beak shape in a representative sample of Asian barbets (Megalaimidae: *Psilopogon*). Specimens photographed are held in the collections of the Smithsonian National Museum of Natural History (USNM), Washington DC, USA. The scale bar represents 1 cm.

**Figure 2.**
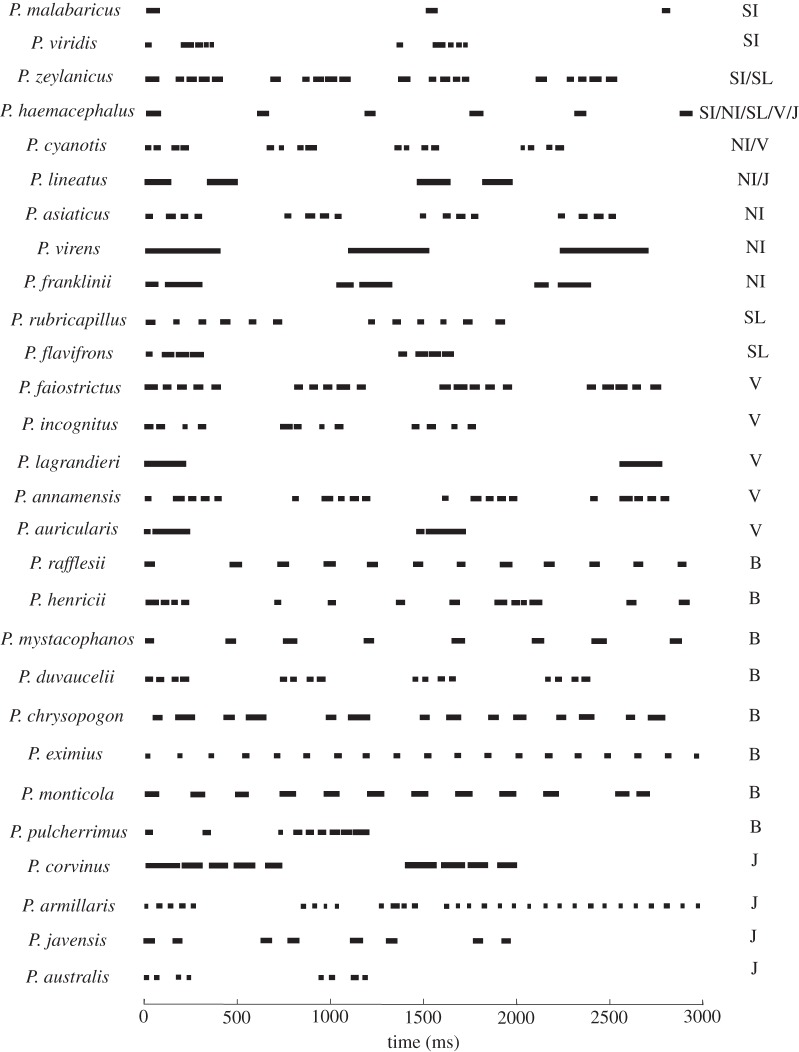
Temporal structure of barbet vocalizations. Shown here are the calls of 28 species from sympatric communities across Asia. Note that each species possesses a stereotyped phrase structure, as well as a high repetition rate of vocalizations. SL, Sri Lanka; SI, South India; NI, Northeast India; V, Vietnam; B, Borneo; J, Java.

Our study focused on Asian barbets (Aves: Megalaimidae), of which 35 species [45] occur across Asia. Two of these (the basal genus *Caloramphus*) are social birds that forage both in the understory and canopy of forest; the other 33 (the genus *Psilopogon*) are territorial canopy frugivores [45]. The latter are ideal candidates to address the questions outlined earlier, for multiple reasons: first, they occur across their range in multispecies assemblages, with up to seven species co-occurring in places [46,47]. Second, they communicate with repetitive advertisement calls (described as songs in Horne & Short [47]), vocalizing throughout the day (and sometimes even at night). Barbets are non-passerines, and are not thought to learn their vocalizations; vocal repertoires consist of repetitive phrases given both within and outside the mating season by both sexes [46–49]. This, together with their occurrence in multispecies assemblages, renders them good subjects for a study of acoustic signalling and avoidance of acoustic competition. Third, the genus shows considerable interspecific morphological variation (figure 1); body sizes range from 34 (*P. eximius*) to 295 g (*P. virens*) [46]. Because body size influences the life histories of organisms [8], this variation may indicate niche divergences within the family. Finally, the phylogeny of this family has recently been elucidated [50], thus enabling study of the evolutionary patterns of traits. We first investigated the phylogenetic structure of barbet communities, together with community organization in both morphological and acoustic traits. The duration of a phrase varies between barbet species [48], with species-specific temporal patterning within each repeating phrase [46,47]. This temporal patterning is diverse and complex; some species possess irregularly spaced elements (individual notes) within a phrase, others groups of evenly spaced elements, and still others use single repetitive elements (figure 2). Importantly, however, the phrase itself is a stereotyped unit that is repeated again and again with relatively little variation [46]. The high repetition rate of calls, in addition to the fact that multiple species vocalize simultaneously over large portions of the day [46], reduces the effectiveness of temporal mechanisms in reducing masking interference [24,51]; in that case spectral differences assume greater importance [52,53]. We therefore investigated if sympatric communities of barbets were divergent in their vocal frequencies. Finally, we investigated the patterns of phylogenetic signal in these traits that may have contributed to community organization. Our data suggest that diversification of morphological traits is important in enabling multiple species to coexist. Because body size and vocal frequency are negatively correlated in barbets [48], we hypothesize (based on trait patterns and community structure) that vocal frequency differences between sympatric species are an indirect by-product of their divergent morphologies.

## 2. Material and methods

### 2.1. Barbet communities

To determine the species composition of sympatric barbet communities, we first obtained occurrence data (based on both museum specimens and sightings) from the Global Biodiversity Information Facility (GBIF; http://www.gbif.org) for six regions across Asia. These regions were selected based on diversity and endemism of barbet species, as well as a relatively high number of records, being well visited by ornithologists and birdwatchers. These regions (with GPS coordinates) were: Southwest (11.07°N, 75.65°E to 13.13°N, 78.0°E) and Northeast (26.68°N, 92.17°E to 27.77°N, 93.70°E) India, South Vietnam (11.75°N, 108.32°E to 12.11°N, 108.7°E), Borneo (Sabah) (5.54°N, 116.37°E to 6.24°N, 116.8°E), Southwest Sri Lanka (7.05°N, 80.41°E to 7.48°N, 80.93°E) and West Java (7.17°S, 105.91°E to 6.11°S, 106.95°E). Occurrence data were then imported into QGIS (Quantum GIS Development team 2013; http://qgis.osgeo.org) and mapped. To determine which barbet species coexisted in the same habitat, we sorted geographically overlapping species into sympatric communities based on the occurrence data, as well as information on local-scale co-occurrence from published bird surveys [54–58]. In addition, we used information about the altitudinal range and habitat preference of each species to improve our resolution of the patterns of co-occurrence [46,47,59]. For example, *P. haemacephalus* and *P. rubricapillus/P. malabaricus* co-occur geographically in parts of Sri Lanka and South India, respectively, and occupy similar altitudinal ranges, but are separated by habitat, with *P. rubricapillus* and *P. malabaricus* tending to replace *P. haemacephalus* in wet evergreen habitats. In addition, many species tend to separate altitudinally when co-occurring. Examples of this include *P. franklinii* and *P. asiaticus* in India, and *P. annamensis, P. auricularis* and *P. incognitus* in Vietnam*.* We note here that although *P. lineatus* and *P. haemacephalus* also occur in Vietnam, these populations are not subspecifically distinct from those in India, and also occupy less dense habitats than other geographically co-occurring species. Because we have already investigated the trait patterns in these taxa in the Indian community, we have not included them in our figures of the Vietnamese community.

### 2.2. Phylogenetic structure of barbet communities

To determine the phylogenetic structure of barbet communities, we tested if they were composed of closely related species using a published phylogenetic tree constructed from mitochondrial and nuclear DNA sequence data [50]. We used the comm.phylo.cor function in the R [60] package ‘picante’ [61,62] that first calculates the pairwise phylogenetic distance (cophenetic distance) between species and then correlates this with an index of co-occurrence to obtain a metric of phylogenetic structure [3,63–65]. We calculated a co-occurrence index for each species pair in four barbet communities by dividing the regions into 0.2 × 0.2° grid cells (518 records in total). We determined the presence/absence of each barbet species in each cell, constructed a presence (1)–absence (0) matrix of co-occurring species for each cell with records of barbets, and pruned the phylogeny to include only those species occurring in that region. From the occurrence data, we calculated the DO_*ij*_ index of co-occurrence; this metric is quite robust to potentially confounding differences in species abundance [6]. DO_*ij*_ is calculated as (*P*_*ij*_ − *P*_*i*_*P*_*j*_)/(*P*_*i*_*P*_*j*_), where *P*_*i*_, *P*_*j*_ and *P*_*ij*_ are the proportion of sites containing each of the species and both of them, respectively. Under independent distributions of species, DO_*ij*_ is approximately equal to zero [6]. We did not calculate correlations for Sri Lanka and Vietnam, because the relatively low number of records in GBIF would have resulted in inaccurate coefficients.

The comm.phylo.cor function also created randomized null communities using the ‘independentswap’ algorithm (999 replicates) [66] to compare with the observed data. This algorithm was selected as it preserves both the frequency of occurrence of each species, and the overall species richness of communities. By comparison with this null community, we could determine if communities exhibited significant phylogenetic structure [4,6,66].

### 2.3. Morphological measurements from museum skins

We performed morphological measurements on 329 museum skins representing 34 of the 35 species of Asian barbets, held in the extensive collections of the United States National Museum of Natural History (USNM) in Washington DC, and the American Museum of Natural History (AMNH) in New York (electronic supplementary material, data file S1). For each specimen, we quantified seven morphological characters: beak length (exposed culmen), beak width and depth (measured at the location of the nares), tail and tarsus length, whole body length and wing chord length, traits typically used in studies of avian community organization [67]. Wherever possible, we measured specimens of both sexes, as well as from across the geographical range of each species. Care was taken to select specimens that were in good condition for measurement (e.g. with beaks intact), so that there were no gaps in our morphometric data. We then log-transformed each individual measurement to linearize the allometric scaling relationships between traits, and to achieve a multivariate normal distribution [67–72]. Following this, we performed a principal components analysis (PCA) in Matlab (MathWorks Inc, Natick, MA) (using a singular value decomposition algorithm) to reduce dimensionality of our multivariate dataset, and thus ordinate the trait morphospace over a reduced number of axes [68,69]. In order to determine the extent of morphospace overlap of species within a community, we calculated pairwise measures of Cohen's *d* for each geographically overlapping species pair. Cohen's *d* is a standardized measure of the overlap between distributions, which takes into account their standard deviations (‘effect size’). This statistic, therefore, allowed us to factor intraspecies variability into the measures of trait overlap. To be conservative, we considered species to overlap in trait space if Cohen's *d* was less than 2 for both PC1 and PC2 (i.e. the difference between species means was less than twice their pooled standard deviation). For species with sample size less than 3, we did not calculate Cohen's *d*; descriptions of their traits are qualitative only (*d*-values for all traits are in electronic supplementary material, data file S2). In order to determine whether the distribution of traits in each community differed from a random draw of traits, we constructed a randomized ‘null’ distribution by randomly selecting trait (PC1) values from the pool of values for the entire family (while maintaining the same community size) 10 000 times. For each geographic region, we calculated the average species dissimilarity (mean pairwise Cohen's *d*) to obtain a distribution of 10 000 values. We then determined the *Z*-score of the observed trait values (mean pairwise Cohen's *d* for each community) with respect to this ‘null’. In order to obtain a more reliable *Z*-score, we repeated this calculation 100 times (each time recalculating the ‘null’ distribution), and calculated the mean *Z*-score from these 100 replicates.

### 2.4. Acoustic trait analyses

To quantify the vocal frequencies of barbets, we analysed call recordings from online databases (Xeno-Canto (http://www.xeno-canto.org) and AVoCet (http://avocet.zoology.msu.edu); electronic supplementary material, tables S1–S2), using the bioacoustics software Luscinia [72]. For each recording, we analysed at least 10 complete phrases. Some recordings were shorter than this; in those cases, we analysed all the calls in the recording. Luscinia created spectrograms of each sound file with which we could delineate the time duration of each vocal element, using an on-screen cursor. After marking the requisite number of phrases on the spectrogram, we used the analysis routines inbuilt in Luscinia to calculate the peak frequency (the frequency of highest amplitude [24]) for each element. In order to calculate the range of vocal frequencies for each species of *Psilopogon* within a community, we tried, where possible, to analyse sound recordings where only one individual of a given species was calling, without any other conspecifics or heterospecifics (electronic supplementary material, table S1). As a result, the sample size for some species was low. Therefore, in order to verify our findings using a different method (and also to account for variability in recording equipment, a caveat inherent in collecting disparate data from song databases), we additionally analysed 37 recordings where more than one species was calling simultaneously (spanning a total of 23 species of barbet, electronic supplementary material, table S2), to compare their vocal frequencies with each other. Most of these recordings involved two simultaneously calling species, but several involved three, and, exceptionally, up to five simultaneously vocalizing species. The identity of these heterospecifics, if not listed on the database catalogue entry for the recording, was determined by comparison with other recordings in the database.

For the within-community analyses (single species recordings), the average of each recording constituted a single sample. We quantified frequency overlap between species using Cohen's *d* and comparison with a randomized draw of traits as detailed earlier, adopting the same criteria for the *d*-value and sample size as outlined in the morphometric analyses. When analysing calls from two or more species in the same recording, we took care to only choose calls that did not overlap in time with each other. This allowed more reliable estimates of peak frequency, although the number of analysable calls was lower. In this case, because we were comparing species within a recording, we used each element of a phrase as a unit in statistical analyses. Because the number of simultaneously vocalizing species varied from two to five across recordings, we performed a statistical test for each recording separately, as opposed to a single paired test for the entire dataset. Where there were two species vocalizing together, we compared the frequencies of both using two-tailed Mann–Whitney *U*-tests, and where there were more than two, we used an ANOVA with *post hoc* Bonferroni correction to identify pairwise differences between simultaneously vocalizing heterospecifics.

### 2.5. Phylogenetic signal in traits

We calculated Blomberg's *K* [73] for both morphological traits and peak vocal frequency (average value of each of these traits for each species), to understand the patterns of phylogenetic signal in both acoustic and morphological traits. This analysis sought to clarify whether closely related species resembled each other or differed from each other in traits when compared with a random Brownian model of trait evolution. Phylogenetic signal is the tendency of related taxa to more closely resemble each other owing to more recent shared ancestry. Blomberg's *K* is a ratio of mean squared errors; *K* < 1 implies that related species are more divergent than expected under a Brownian model of evolution, whereas a *K* > 1 implies the converse [74]. Pagel's lambda is also commonly used to detect phylogenetic signal in traits, but being a multiplicative factor, it may not be as accurate as the *K*-statistic. In addition, the value of lambda under Brownian motion is 1, and there is no definition for lambda above 1, which limits its utility in detecting phylogenetic constraints in a trait [75]. To generate a randomized null tree to compare with the observed value of *K*, we shuffled the tips of the tree randomly 1000 times (breaking the association between species and phylogenetic position to generate a true random tree with no clustering of tips). The significance (*p*-value) of *K* was determined by comparing the observed variance in trait values (across phylogenetically independent contrasts) to those of the null distributions.

## 3. Results

### 3.1. Sympatric co-occurrence in barbets is not correlated to their phylogenetic relatedness

First, we investigated the phylogenetic structure of barbet communities. In four of our sympatric communities (518 records in total), the metric of phylogenetic structure (correlation between co-occurrence and phylogenetic relatedness) did not differ significantly from a randomized null community (results in table 1). Thus, sympatric assemblages appear to be drawn randomly from across the phylogeny, and analysis of phylogenetic structure by itself did not show signatures of either environmental filtering or competition.

**Table 1.** Phylogenetic relatedness does not correlate with species coexistence (DO_*ij*_) in communities of Asian barbets. Sympatric assemblages are neither phylogenetically conserved nor overdispersed. The correlation represents a metric of phylogenetic structure within communities, and is not statistically significant; nor does it differ from a randomly generated null community.

region	number of species in region	correlation coefficient (DO_*ij*_ versus phylogenetic relatedness)	*p*-value for correlation coefficient	*p*-value for randomization test
South India	4	0.106	0.841	0.077
Northeast India	6	−0.316	0.374	0.177
Borneo	8	−0.115	0.683	1
Java	6	0.123	0.662	0.423

### 3.2. Morphospace separation in sympatric Asian barbets

**Figure 3.**
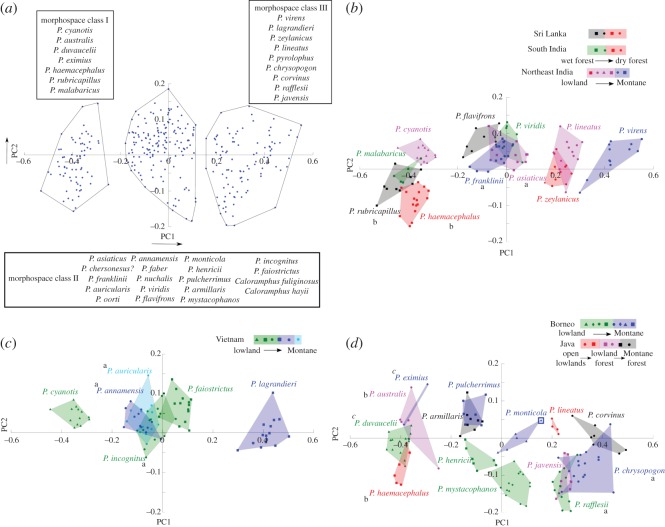
Morphological divergence in sympatric Asian barbets. (*a*) Plot of first (PC1; *x*-axis) versus second (PC2; *y*-axis) principal component scores for seven morphological characters (*n* = 329). Asian barbets sort into three distinct morphospace classes (list of species in the boxes), corresponding to large-, medium- and small-sized species. One species missing from our dataset is marked ‘?’, and is assigned tentatively to morphospace class based on comparison with published literature. (*b–d*) Trait morphospace of sympatric barbets from: (*b*) India and Sri Lanka, (*c*) Vietnam and (*d*) Borneo and Java. The symbols represent different species, and the key to the top right of each plot shows the species composition of each sympatric community, along with their habitat preferences. The background colours in the key and the polygons unite species that co-occur in similar habitats; species on the boundary between two colours in the key may occur in both habitats. Each species is represented by samples from across its geographical range, including from outside the region in question. For *P. haemacephalus* and *P. lineatus*, the endemic subspecies from Java are plotted separately in (*d*), whereas mainland Asian subspecies are plotted in (*b*). For a list of specimens measured, see electronic supplementary, data file S1. The letters a, b and c next to species names denote species groups in each region that we determined to overlap in PC1 and PC2 morphospace. (Cohen's *d* < 2 for both PC1 and PC2; the ‘c’ in plot (*d*) is italicized because our sample size was not sufficient for *P. eximius* to determine Cohen's *d*. We note, however, that it is qualitatively closest to *P. duvaucelii*, and may overlap with it). Note that these overlapping species are typically separated by habitat or altitude. (A single specimen of *P. monticola*, USNM 328036, grouped with morphospace class III, whereas the other five specimens measured grouped with class II. The data point for this specimen is marked using a square with a blue boundary in (*d*) to distinguish it from other specimens of the same species. This specimen is also larger than measurements in the literature [47]; therefore, we have provisionally placed *P. monticola* in morphospace class II (*a*), pending further study.)

To draw inferences about the mechanisms of community assembly, we proceeded to study trait patterns in sympatric barbets across Asia. The first two principal components of our morphometric dataset (abbreviated as PC1 and PC2) together explained approximately 95% of the variation in the data (table 2). We performed a morphospace ordination by plotting PC1 and PC2 scores against each other [67–69]. The 34 species we examined sorted into three distinct clusters along the PC1 axis (figure 3*a*), which we refer to as morphospace classes I, II and III henceforth for convenience. Classes I and III contained seven and nine species, respectively, with the remainder falling in the intermediate class II. Comparing the species in each class (figure 3*a*) with the original measurements (electronic supplementary material, data file S1), larger-bodied species tended to have higher PC1 scores, and grouped in class III, whereas the smaller species in the family all grouped in class I, and had lower PC1 scores (also see figure 3*b–d*). This suggests that morphospace classes I, II and III correspond to small, medium and large size classes. In general, all specimens of a given species grouped with a single morphospace class including geographical and gender variation, with a single specimen of *P. monticola* being the sole exception (figure 3*d*, also see figure caption).

**Table 2.** Results of principal components analysis for seven morphological characters. Rows contain factor loadings for each trait, eigenvalues and the proportion of variance explained by each principal component.

	PC1	PC2	PC3	PC4	PC5	PC6	PC7
beak length	0.4872	−0.3645	0.0461	−0.1704	0.6829	−0.3559	−0.0745
beak width	0.323	−0.5384	0.3416	0.3626	−0.5606	−0.2073	−0.0194
beak depth	0.3887	−0.2424	−0.4243	0.2336	0.0743	0.734	0.1057
tail length	0.4423	0.6604	0.1529	0.5366	0.0972	−0.0791	−0.2029
tarsus length	0.2764	0.1343	0.6599	−0.5112	−0.0645	0.4518	−0.0219
body length	0.3807	0.2387	−0.2284	−0.2542	−0.2445	−0.2565	0.7455
wing length	0.3004	0.0854	−0.436	−0.4135	−0.3749	−0.1239	−0.6209
eigenvalue	0.0694	0.0054	0.0012	0.0011	0.001	0.0006	0.0004
% explained variance	87.7794	6.8217	1.5738	1.3303	1.2218	0.8078	0.4652
cumulative %variance	87.7794	94.6011	96.1749	97.5052	98.727	99.5348	100

We next compared the morphospace patterns in sympatric communities of barbets across Asia (figure 3*b–d*). In a given geographical region, the local barbet assemblage typically consisted of a mix of species from all three morphospace classes (i.e. large, medium and small species). Nearly all geographically overlapping species occupied distinct regions of PC1–PC2 morphospace from each other, indicating divergent morphologies (Cohen's *d* > 2 for either PC1 or PC2, *d*-values in electronic supplementary material, data file S2, sample sizes in electronic supplementary material, data file S1). Geographically overlapping species with the most morphospace overlap (*d* < 2 for both PC1 and PC2) were as follows: *P. rubricapillus/P. haemacephalus* and *P. asiaticus/P. franklinii,* (b and a in figure 3*b*, respectively), *P. annamensis* and *P. auricularis/P. incognitus,* (a in figure 3*c*), *P. rafflesii/P. chrysopogon* and *P. haemacephalus/P. australis* (a and b in figure 3*d*, respectively; also bold values in electronic supplementary material, data file S2). Additionally, although our sample size for *P. eximius* was too low to calculate Cohen's *d*, we note that it is qualitatively close in morphospace to *P. duvaucelii* (figure 3*d*, denoted by italicized c). All the above species, however, are separated either ecologically or by altitude, thus minimizing contact with each other (as indicated by the colour coding in figure 3) [46]. We also compared within-community trait distributions (PC1 scores) to a randomized trait distribution (see Material and methods). All six communities were either significantly different from a random distribution (S India: *Z* = 2.31, NE India: *Z* = 2.24, Vietnam: *Z* = 1.98, Borneo: *Z* = 2.28, Java: *Z* = 3.36, *p* < 0.05), or close to the threshold for significance at *p* = 0.05 (Sri Lanka: *Z* = 1.81, *p* = 0.07).

### 3.3. Sympatric barbets vocalize at distinct frequencies

**Figure 4.**
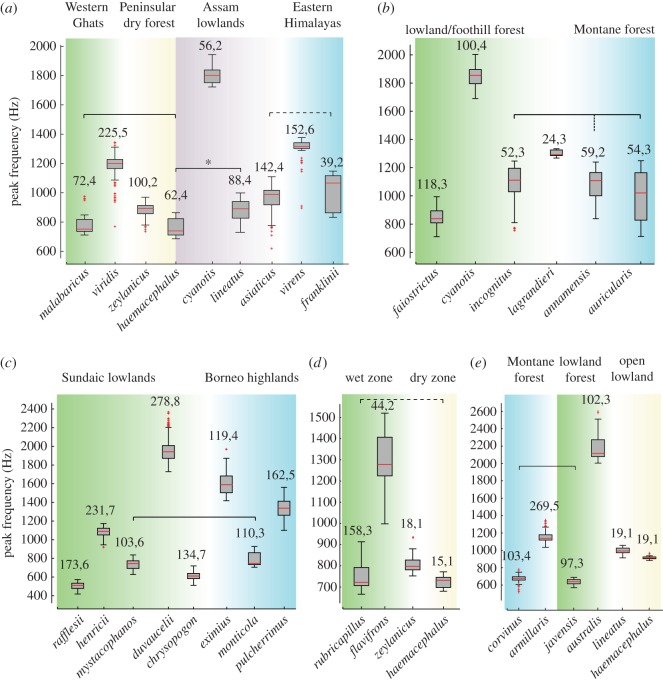
Sympatric Asian barbets are divergent in their vocal frequencies. Box plots represent peak vocal frequencies of barbets from (*a*) India (*b*) Vietnam (*c*) Borneo (*d*) Sri Lanka and (*e*) Java. Species shown at the boundary between two habitats may occur in both. Note that species with similar vocal frequencies are typically separated by habitat or altitude. The numbers separated by commas above each boxplot indicate sample sizes, the left-hand one the total number of song elements analysed (and plotted) and the right-hand side the number of recordings. For a full list of recordings analysed, with locality information, see electronic supplementary material, table S1. The solid lines between species on plots denote species that overlap in vocal frequency (Cohen's *d* < 2), whereas the dashed lines denote species where the number of samples was too low to determine Cohen's *d*, but for which we qualitatively assume overlap based on the distribution of frequencies (in these cases, plotted here for visual comparison). Note that these overlapping species are typically separated by habitat or altitude (the exception being *P. haemacephalus* and *P. lineatus* in (A), marked with a *, which, however, had a *d* = 1.98, so were very close to our threshold).

In order to understand acoustic trait patterns in sympatric communities, we calculated the peak vocal frequencies of barbet vocalizations (124 total recordings from 28 species, see electronic supplementary material, table S1 for a full list organized by species). Sympatric barbets generally vocalized in distinct frequency bands from each other (Cohen's *d* > 2, *d*-values in electronic supplementary material, data file S2, sample sizes are the number of recordings shown in figure 4). For some species, the sample size was too low to accurately determine Cohen's *d*. We note, however, that most of these species exhibit vocal frequencies that are qualitatively distinct from other sympatric species (i.e. species with the same background colour in figure 4). Species with overlapping geographical ranges, which we determined to have overlapping vocal frequencies (*d* < 2) were as follows: *P. haemacephalus*/*P. malabaricus* and *P. haemacephalus/P. lineatus,* figure 4*a*; *P. auricularis/P. incognitus,* figure 4*b*; *P. mystacophanos/P. monticola,* figure 4*c*; and *P. corvinus/P. javensis,* figure 4*e* (represented in the figures by solid lines between species, also bold values in electronic supplementary material, data file S2). Additionally, we also note that *P. franklinii* overlaps qualitatively with *P. asiaticus* in figure 4*a*, *P. annamensis* with *P. auricularis/P. incognitus* in figure 4*b* and *P. rubricapillus* with *P. haemacephalus* in figure 4*d*, although sample sizes were too low to calculate *d* (dashed lines in figure 4). Similar to the morphological traits, these overlapping species are separated either by habitat or by altitude (see background colours on plots) [46,47]. The one exception to this was *P. haemacephalus* and *P. lineatus*, which are sympatric in NE India (asterisk in figure 4*a*). The *d*-value for these two species, however, was 1.98, which was very close to our conservative threshold for overlap, and higher than the *d*-values for other overlapping species (see electronic supplementary material, data file S2). We also performed a randomization analysis (see Material and methods) for four communities with sufficient species data to calculate Cohen's *d*. Trait distributions in three of these four communities differed significantly from a random distribution (Borneo: *Z* = 2.89, Java: *Z* = 2.2, *p* < 0.05, Vietnam: *Z* = 1.92, *p* = 0.054). The Northeast Indian community did not differ significantly from a random distribution (*Z* = 1.03, *p* = 0.3). It is worth noting here that this community contained the exception noted above, as well as that this analysis was conservative in considering only geographical overlap and not local sympatry.

**Figure 5.**
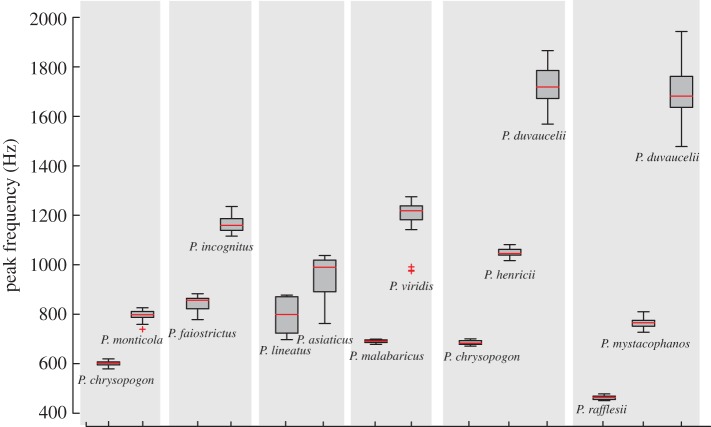
Simultaneously vocalizing barbet species occupy distinct frequency bands. Shown here are six representative examples (of 37 total analysed, see electronic supplementary material, table S2 for the full dataset) of peak frequency separation when multiple species vocalize together (hash symbol in electronic supplementary material, table S2). Each grey bar represents one example recording. In every single case (of 37 total), the vocal frequency of each species was statistically distinguishable from all other simultaneously vocalizing species (*p* < 0.01). See electronic supplementary material, table S2 for the results of statistics in each case.

To further verify these findings, we also analysed 37 recordings of two to five barbet species vocalizing simultaneously (six representative examples shown in figure 5, see electronic supplementary material, table S2). Consistent with the previous finding, barbets invariably vocalized at significantly different peak frequencies from simultaneously signalling heterospecifics (*p* < 0.01, Mann–Whitney *U*-test for comparison of two species, ANOVA with *post hoc* Bonferroni correction for more than two species; statistics and sample sizes in electronic supplementary material, table S2). In recordings with more than two species, *post hoc* analyses revealed significant (*p* < 0.05) differences between each pair of vocalizing species.

### 3.4. Phylogenetic patterns in morphological and acoustic traits

Based on the combination of random phylogenetic dispersion in communities, and the patterns of trait divergence, we predicted that the traits that are more important in community assembly would show signals of divergence between related species [3]. Blomberg's *K* (a measure of phylogenetic signal) was significant (*p* < 0.001, 37 taxa) for all traits measured when compared with a randomized tree. For vocal frequency, the value of *K* was close to 1 (0.97), indicating that phylogenetic patterns in this trait were close to those expected under a Brownian model of evolution (see Material and methods), where divergences occur over time in a random walk [76]. *K*-values for morphological principal component scores (table 3, as well as the individual traits, electronic supplementary material, table S3), on the other hand, were lower than 1. Thus, closely related species were more morphologically divergent than expected under Brownian evolution, indicating signatures of morphological diversification in the evolution of barbets.

**Table 3.** Phylogenetic signal in morphological traits and vocal frequencies of Asian barbets (37 taxa, including species and subspecies, as per the consensus tree in [50]). The *p*-values indicate the significance of the estimated value of *K* when compared with a randomized null tree, whereas the *K*-value itself indicates whether or not phylogenetic signal follows a Brownian model of evolution (see Material and methods for more details). Note that while vocal frequency does not show a difference from a Brownian model of evolution (*K* close to 1), morphological PC scores (as well as individual traits, see electronic supplementary material, table S3) show a *K* < 1, indicating that related species are more divergent morphologically than expected under Brownian evolution.

trait	Blomberg's *K*	*p*-value
morphometric PC1 score	0.4936223	<0.001
morphometric PC2 score	0.231594	<0.001
peak vocal frequency	0.9762429	<0.001

## 4. Discussion

Our investigations of community structure and trait patterns in barbet assemblages across Asia revealed that barbet communities are phylogenetically randomly dispersed. Thus, phylogenetic structure alone did not reveal strong signatures of either competitive interactions or environmental filtering. However, we also found that across communities, sympatric species are divergent in both morphological and acoustic space. In the following section, we discuss trait divergence in the context of the natural history of barbets, and putative mechanisms of community assembly by matching our findings to the predictions laid out in previous studies of community phylogeny [3,4].

### 4.1. Morphological diversification and ecological niche divergence in Asian barbets

Character displacement or the divergence of traits in sympatry owing to direct competition, has been identified as underlying many examples of niche divergence [9,10,25,30]. Niche divergence may also result through evolutionary drift or ecological speciation in allopatry; in that case species already occupy separate niches if their ranges subsequently overlap [77]. In this scenario, competition does not directly influence trait evolution, but communities are still composed of species with divergent traits. We find that Asian barbets form three morphological classes, which broadly correspond to large-, medium- and small-sized species based on a combination of bill and body characters. This morphological structuring parallels the well-studied ecological niche divergence of Darwin's finches (*Geospiza*) which also group into large, medium and small size classes [13]. Similarly, Wallacean horseshoe bats exist as three sympatric size morphs, which vocalize at three distinct harmonic frequencies [28]; the largest morph uses the lowest frequency, a pattern also seen in tinkerbirds [25]. We also find that sympatric barbets occupy distinct regions of morphospace; communities typically consist of small-, medium- and large-sized species, which further supports the idea that morphological diversification is a result of ecological niche divergence. Niche diversification may occur in sympatry or allopatry, with subsequent sympatry being contingent on reduced niche overlap [77]. Whereas bill traits are directly related to food resource use (bill size constrains the size of fruit the bird can consume), body traits relate to diverse aspects of life history from aerodynamics to habitat use [78]. Although the ecology of many barbet species is poorly understood, several further lines of evidence suggest niche divergence between sympatric barbets. Barbets in Sundaland partition food resources by size, with larger species taking larger figs [79], and may show dominance hierarchies at fruiting trees [46]. As cavity-nesters, barbets also compete for scarce nesting and roosting sites, and may eject heterospecific eggs and nestlings [46]. *P. faiostrictus* and *P. incognitus* have been observed sharing a nesting tree, however, suggesting that, like the feeding niche, there may be also some interspecific differentiation in nest site selection [46]. Divergent beak and head morphologies (figure 1) may allow species to exploit different types of wood for nesting (as in woodpeckers) [80]. This is, however, purely speculative at this point, and requires further study. Finally, morphologically similar species are often altitudinally (*P. franklinii/P. asiaticus*), or ecologically (*P. haemacephalus*/*P. rubricapillus*) separated [46]. Together with our findings, this indicates that morphologically divergent species coexist owing to reduced niche overlap. Competitive interactions between similar species may lead either to trait divergence or competitive exclusion, thus resulting in divergent traits within communities. Short & Horne [46] suggested that the distributions of many barbet species (particularly in Sundaland) indicate allopatric speciation followed by subsequent competitive exclusion, which supports the latter scenario. However, caution must be exercised in drawing mechanistic inferences from current trait distributions within communities. It is possible that both character displacement and competitive exclusion may influence trait structure within communities, particularly where the ranges of morphologically similar species abut (e.g. *P. lineatus* and *P. zeylanicus* in Northern India) [46]. Additionally, interactions with other sympatric frugivores (such as hornbills and pigeons) may also influence the traits and life histories of barbets; the ecology of sympatric canopy frugivores thus also merits further study.

### 4.2. Vocal frequency divergence and acoustic signalling

Differentiation of acoustic signals serves variously to avoid masking interference, and to discriminate species from each other. Maintaining distinctness within a vocalizing chorus facilitates territorial advertisement and mate attraction [18,81,82], and may serve as a reinforcing signal to maintain reproductive isolation [11]. As mentioned earlier, acoustic signal divergence may occur in both the temporal and frequency domains. Frogs are a well-studied example of acoustic signal partitioning in sympatric communities; species partition physical, temporal and frequency space to avoid overlap with each other [24,42,83,84]. A growing body of literature in both frogs and crickets has highlighted frequency differences as being important in discriminating sounds in heterospecific choruses [19,24,52,81]. In both these animals, acoustic signals are stereotyped and rapidly repeated, thus reducing the effectiveness of temporal divergence in avoiding masking [24,51]. Divergent frequencies may thus play a greater role in avoiding masking. In psychophysics experiments, starlings can perceptually segregate two sound streams based only on frequency differences [85].

The acoustic signals of Asian barbets are a prominent feature of their life histories [46]. Both sexes vocalize [49], and it has been suggested that this serves functions of communication and territorial advertisement [48]. In addition to the striking patterns of morphological diversification, we found that coexisting barbets exhibit divergent vocal frequencies. Where species with similar vocal frequencies (and also similar morphologies) co-occur geographically, they are typically separated either by altitude or habitat. In this regard, we observe similarities between barbet assemblages and those of frogs and crickets [20,24,42] in contrast to passerine birds, which often exhibit temporal partitioning or changes in structural variability of songs [23,34,35,86]. This may relate to the fact that barbets, like frogs and crickets, possess relatively rigid, stereotyped song, unlike the flexible repertoires of passerine birds, which learn their songs.

Finally, the role of visual signals in species recognition is important to discuss, given the presence of species-specific coloured patterns on the heads of barbets [46,48]. Visual signals promote differentiation when acoustic cues are similar [87], but are likely to be more effective at short ranges [88]. Barbets of the genus *Psilopogon* are cryptic in the forest canopy, which supports a role for visual signals in short-range recognition [46]. Divergent vocal frequencies may thus facilitate long-range communication by minimizing masking interference.

### 4.3. Divergent vocal frequencies may be an indirect consequence of divergent morphological traits

To summarize, we find that sympatric barbets are divergent in both morphological traits and vocal frequencies, and also that they are phylogenetically randomly dispersed. Cavender-Bares *et al*. [3] predict that this scenario may occur if competitive interactions (or competitive exclusions) cause the structuring of traits that are divergent between related species. Therefore, the traits that are important determinants of species composition in communities should show evolutionary signatures of such a divergence. We find phylogenetic signatures of pronounced morphological divergence between closely related species, more so than expected under Brownian evolution (Blomberg's *K* < 1). Vocal frequency divergences, on the other hand, are close to patterns expected under Brownian evolution, which is in agreement with other studies [48,89,90]. By matching these patterns to the predictions outlined above, we thus hypothesize that morphological divergence is key in allowing multiple species to coexist, and that the divergent vocal frequencies of sympatric barbets are a by-product of their divergent morphologies. Vocal frequency in barbets correlates negatively with body size [48], which supports this hypothesis. Additionally, beak shape also influences vocal frequency in passerines [91–93], presenting another potential influence of morphology on vocal signals. Barbets typically vocalize with their beaks closed or nearly so [46]. Air being forced through this narrowed aperture may also influence vocal acoustics, for example bandwidth and tonal qualities of sound [94]; this will be the focus of future studies.

Kirschel *et al*. [25] identified patterns of character displacement in both morphology and vocal frequencies of two sympatric tinkerbird species (Lybiidae: *Pogoniulus*), African barbets which are close relatives of the Megalaimidae. Character displacement followed the relationship described above (i.e. a negative relationship between size and frequency). These authors suggested that reproductive interference was the likely cause of this character displacement. Our study of Asian barbets investigated similar phenomena at the level of communities. We suggest that morphologically divergent species coexist owing to reduced niche overlap, and that this indirectly leads to divergent vocal frequencies within communities. Similar mechanisms of community assembly may operate in diverse animals, including other birds that do not learn their vocalizations. Studies that combine trait structure, behavioural and phylogenetic analyses may thus be fruitful in understanding community-level processes influencing sensory signal evolution.

## Supplementary Material

Barbet_Krishnan Tamma Supplementary Material_v19_R4_final.doc - Title: Supplementary Tables. Description: Supplementary Tables S1-S3.

## Supplementary Material

Barbet_Krishnan_Tamma_2015_SuppFile1v19_R2.xls - Title:List of specimens measured, and morphological measurements. Description: Specimens used in morphospace analyses.

## Supplementary Material

Barbet_Krishnan_Tamma_2015_Supp File2v19_R2.xlsx - Title: Measures of Cohen's d for morphological and acoustic traits. Description: Quantification of trait overlap for data in Figures 3 and 4.

## Permission to carry out fieldwork

No fieldwork was conducted as part of this study.

## Data accessibility

The supplementary material contains the information necessary to locate the recordings used in our study, which were sourced from online databases (see Material and methods for links).
