# Supplementary material for: Divergent morphological and acoustic traits in sympatric communities of Asian barbets
Source: R Soc Open Sci. 2016 Aug 10;3(8):160117. doi: 10.1098/rsos.160117 (PMC5108939; doi:10.1098/rsos.160117)
Supplement: Barbet_Krishnan Tamma Supplementary Material_v19_R4_final.doc - Title: Supplementary Tables. Description: Supplementary Tables S1-S3. [file rsos160117supp1.doc]

**Supplementary Tables: Divergent Morphological and Acoustic Traits in Sympatric Communities of Asian Barbets**

**Anand Krishnan^1, *^ and Krishnapriya Tamma^2, *^**

**Author Affiliations:**

1. **Department of Psychological and Brain Sciences, Johns Hopkins University, Baltimore, MD 21218, USA.**
2. **National Centre for Biological Sciences, Tata Institute of Fundamental Research, GKVK Campus, Bangalore 560065, Karnataka, India.**

***- Equal contribution.**

**For correspondence:**

**Email:** [**akrish16@jhu.edu**](mailto:akrish16@jhu.edu)**,** [**priya.tamma@gmail.com**](mailto:priya.tamma@gmail.com)

**Supplementary Table S1:** Recordings used for the analyses in Figure 4, with locations and recordist attributed. The prefix XC reflects recordings from the Xeno-Canto database, while the prefix AV represents recordings from the AVoCet database. Recordings are arranged according to species, in alphabetical order of specific name.

| Species | Catalog number | Recordist | Location |
| --- | --- | --- | --- |
| *P.annamensis* | XC126655 | Patrik Aberg | Deo Nui San, Vietnam |
|  | XC200947 | Frank Lambert | Dalat Plateau, Vietnam |
| *P.armillaris* | XC40055 | Patrik Aberg | Bali Botanical Gardens |
|  | XC71638 | Bas van Balen | Gunung Halimun, Java |
|  | XC68665 | Frank Lambert | Cibodas, Java |
|  | XC40450 | David Edwards | Gunung Gede, Java |
|  | AV15321 | Bram Demeulemeester | Gunung Gede, Java |
| *P.asiaticus* | XC79720 | Frank Lambert | Digboi Oilfields, India |
|  | XC79747 | Frank Lambert | Nameri NP, Assam, India |
|  | XC96594 | Sudipto Roy | Narendrapur, West Bengal, India |
|  | AV9153 | Pamela C. Rasmussen | Digboi, India |
| *P.australis* | XC70178 | David Gibbs | Carita, Java |
|  | XC70182 | David Gibbs | Ujong Kulon, Java |
|  | AV15702 | Pamela C. Rasmussen | Carita, Java |
| *P.auricularis* | AV5283 | Frank R. Lambert | Mt. Fansipan, Tonkin, Vietnam |
|  | XC200854 | Frank Lambert | Dalat Plateau, Vietnam |
|  | XC200949 | Frank Lambert | Dalat Plateau, Vietnam |
| *P.chrysopogon* | XC158788 | Arend Wassink | Fraser’s Hill, Pahang, Malaysia |
|  | XC166578 | Patrik Aberg | Danum Valley, Sabah, Borneo |
|  | XC173932 | Marc Anderson | Taman Negara, Pahang, Malaysia |
|  | XC196835 | Frank Lambert | Kubah NP, Sarawak, Borneo |
|  | XC203298 | Frank Lambert | Mt. Kinabalu, Sabah, Borneo |
|  | AV14095 | Frank R. Lambert | Selangor, Malaysia |
|  | AV17210 | Julia Miller | Sarawak, Borneo |
| *P.corvinus* | XC18839 | Mike Catsis | Gunung Halimun, Java |
|  | XC70196 | David Gibbs | Gunung Gede, Java |
|  | XC79355 | Desmond Allen | Gunung Gede, Java |
|  | AV13635 | Pamela C. Rasmussen | Gunung Halimun, Java |
| *P.cyanotis* | XC125336 | Patrik Aberg | Cat Tien NP, Vietnam |
|  | XC125337 | Patrik Aberg | Cat Tien NP, Vietnam |
|  | XC200721 | Frank Lambert | Cat Tien NP, Vietnam |
|  | XC26053 | Martjan Lammertink | Alaungdaw Kathapa NP, Myanmar |
|  | XC93970 | Mike Nelson | Kaeng Krachan NP, Thailand |
|  | AV8615 | Abidur Rahman | Kaziranga NP, Assam, India |
| *P.duvaucelii* | XC167094 | Patrik Aberg | Kinabatangan, Sabah, Borneo |
|  | XC184102 | Ding Li Yong | Bentong, Pahang, Malaysia |
|  | XC192436 | Mike Nelson | Taman Negara, Pahang, Malaysia |
|  | XC196833 | Frank Lambert | Kubah NP, Sarawak, Borneo |
|  | XC20213 | Sander Pieterse & Ben Wielstra | Gunung Lumut, East Kalimantan, Borneo |
|  | XC58901 | Frank Lambert | Tanjung Tuan, Malaysia |
|  | XC68668 | Frank Lambert | Danum Valley, Sabah, Borneo |
|  | AV450 | Pamela C. Rasmussen | Sepilok, Sabah, Borneo |
| *P.eximius* | XC23070 | George Wagner | Crocker Range, Sabah, Borneo |
|  | XC28857 | David Edwards | Poring HS, Sabah, Borneo |
|  | XC34887 | Sander Pieterse | Crocker Range, Sabah, Borneo |
|  | AV7327 | Pamela C. Rasmussen | Crocker Range, Sabah, Borneo |
| *P.faiostrictus* | XC165457 | Richard Dunn | Kaeng Krachan NP, Thailand |
|  | XC200723 | Frank Lambert | Cat Tien NP, Vietnam |
|  | AV1795 | P.D. Round | Khao Yai, Thailand |
| *P.flavifrons* | XC164019 | Tero Linjama | Sinharaja, Sri Lanka |
|  | XC183229 | Marc Anderson | Sinharaja, Sri Lanka |
| *P.franklinii* | XC19679 | David Farrow | Mongar, Bhutan |
|  | XC80021 | Frank Lambert | Eaglenest WLS, Arunachal Pradesh, India |
| *P.haemacephalus* | XC166134 | Frank Lambert | Melghat, Maharashtra, India |
|  | XC190048 | Pronoy Baidya | Kalapet, Tamil Nadu, India |
|  | XC44805 | Sander Bot | Fort Aguada, India |
|  | XC183232 | Marc Anderson | Yala NP, Sri Lanka |
|  | XC189660 | Ameya Deshpande | Aurangabad, Maharashtra, India |
|  | AV13108 | Pamela C. Rasmussen | Bali Barat NP, Bali |
| *P.henricii* | XC158658 | Arend Wassink | Taman Negara, Pahang, Malaysia |
|  | XC158662 | Arend Wassink | Taman Negara, Pahang, Malaysia |
|  | XC177745 | Johannes Fischer | Murung Raya, Central Kalimantan, Borneo |
|  | XC192794 | Mike Nelson | Panti, Johor, Malaysia |
|  | XC21035 | Martjan Lammertink | Matan, West Kalimantan, Borneo |
|  | XC30040 | David Edwards | Danum Valley, Sabah, Borneo |
|  | AV15326 | Bram Demeulemeester | Taman Negara, Pahang, Malaysia |
| *P.incognitus* | XC19317 | Craig Robson | Kaeng Krachan NP, Thailand |
|  | XC41761 | John Van der Woude | Khao Yai, Thailand |
|  | AV1796 | P.D.Round | Khao Yai, Thailand |
| *P.javensis* | XC42391 | George Wagner | Ijen, Java |
|  | XC90278 | Fabian Ducry & David Marques | Carita, Java |
|  | AV8351 | Bram Demeulemeester | Carita, Java |
| *P.lagrandieri* | XC161809 | Klemens Steiof | Cat Tien NP, Vietnam |
|  | XC175783 | Frank Lambert | Dalat Plateau, Vietnam |
|  | AV12434 | Filip Verbelen | Cat Tien NP, Vietnam |
| *P.lineatus* | XC125382 | Sudipto Roy | Narendrapur, West Bengal, India |
|  | XC149873 | Niels Poul Dreyer | Nameri NP, Assam, India |
|  | AV5902 | Monirul Khan | Madhupur NP, Bangladesh |
|  | AV9154 | Pamela C. Rasmussen | Nameri NP, Assam, India |
|  | AV12969 | Pamela C. Rasmussen | Bali Barat NP, Bali |
| *P.malabaricus* | XC110819 | Vir Joshi | Goa, India |
|  | XC44835 | Sander Bot | Goa, India |
|  | XC44845 | Sander Bot | Goa, India |
|  | AV13486 | Pamela C. Rasmussen | Thattekad, Kerala, India |
| *P.monticola* | XC167254 | Patrik Aberg | Mt. Kinabalu, Sabah, Borneo |
|  | XC194756 | Stijn De Win | Crocker Range, Sabah, Borneo |
|  | XC38546 | Mike Nelson | Crocker Range, Sabah, Borneo |
| *P.mystacophanos* | XC158666 | Arend Wassink | Taman Negara, Pahang, Malaysia |
|  | XC196836 | Frank Lambert | Kubah NP, Sarawak, Borneo |
|  | XC21037 | Martjan Lammertink | Matan, West Kalimantan, Borneo |
|  | XC37636 | John Van der Woude | Taman Negara, Pahang, Malaysia |
|  | XC39223 | Mike Nelson | Danum Valley, Sabah, Borneo |
|  | AV468 | Pamela C. Rasmussen | Poring, Sabah, Borneo |
| *P.pulcherrimus* | XC150762 | Mike Nelson | Mt. Kinabalu, Sabah, Borneo |
|  | XC28841 | David Edwards | Mt. Kinabalu, Sabah, Borneo |
|  | XC35666 | Arnold Meijer | Mt. Kinabalu, Sabah, Borneo |
|  | XC35667 | Arnold Meijer | Mt. Kinabalu, Sabah, Borneo |
|  | AV10452 | Tim Muhich | Mt. Kinabalu, Sabah, Borneo |
| *P.rafflesii* | XC192786 | Mike Nelson | Panti, Johor, Malaysia |
|  | XC192787 | Mike Nelson | Panti, Johor, Malaysia |
|  | XC196838 | Frank Lambert | Kubah NP, Sarawak, Borneo |
|  | XC196840 | Frank Lambert | Kubah NP, Sarawak, Borneo |
|  | XC21038 | Martjan Lammertink | Matan, West Kalimantan, Borneo |
|  | AV10585 | P.D.Round | Krabi, Thailand |
| *P.rubricapillus* | XC19626 | David Farrow | Udawattakele, Kandy, Sri Lanka |
|  | AV6661 | Deepal Warakagoda | Udahamulla, Colombo district, Sri Lanka |
|  | AV6662 | Deepal Warakagoda | Udahamulla, Colombo district, Sri Lanka |
| *P.virens* | XC115000 | Mike Nelson | Lamperi Royal Botanical Park, Bhutan |
|  | XC163079 | Arend Wassink | Phulchowki, Nepal |
|  | XC21892 | Mathias Ritschard | Shillong, Meghalaya, India |
|  | XC35474 | Arnold Meijer | Eaglenest WLS, Arunachal Pradesh, India |
|  | XC42620 | David Farrow | Deothang, Bhutan |
|  | AV8617 | Abidur Rahman | Eaglenest WLS, Arunachal Pradesh, India |
| *P.viridis* | XC125496 | Sudipto Roy | Molem NP, Goa, India |
|  | XC161224 | Conrad Pinto | Goa, India |
|  | XC192283 | Vivek Puliyeri | Nilambur, Kerala, India |
|  | XC90826 | Ramit Singal | Manipal, Karnataka, India |
|  | AV13467 | Pamela C. Rasmussen | Kerala, India |
| *P.zeylanicus* | XC73065 | Mike Nelson | Varanasi, India |
|  | XC97395 | Vir Joshi | Shoolpaneshwar WLS, India |
|  | XC96487 | David Marques | Sigiriya, Sri Lanka |

**Supplementary Table S2:** Recordings of multiple barbet species vocalizing together, with locations and recordist attributed. Average measured peak frequencies are also given in the table, along with the standard error of the mean and the sample size n (i.e. the total number of distinct song elements analyzed). Test statistics are indicated; for recordings with two species, the value of U after a two-tailed Mann-Whitney U-test is provided, while for recordings with more than two species, the F-statistic after an ANOVA test is indicated. All recordings showed a significant difference in peak frequencies between vocalizing species with a p<0.01. A # indicates that the recording is shown as a representative example in Figure 5.

| Catalog number | Species | Peak frequency ± SEM (Hz) | n | Test statistic value | Recordist | Location |
| --- | --- | --- | --- | --- | --- | --- |
| XC93993 | *P.asiaticus* | 1082.99±23.79 | 15 | U=0 | Mike Nelson | Kaeng Krachan, Thailand |
|  | *P.cyanotis* | 1838±13.06 | 17 |  |  |  |
| XC156588 | *P.chrysopogon* | 616.57±7.39 | 19 | U=342 | Marcus Braun | Poring, Sabah, Borneo |
|  | *P.duvaucelii* | 2029.2±21.32 | 18 |  |  |  |
| XC166578 # | *P.chrysopogon* | 684.35±2.35 | 16 | F=1758.75 | Patrik Aberg | Danum Valley, Sabah, Borneo |
|  | *P.duvaucelii* | 1726.7±20.85 | 16 |  |  |  |
|  | *P.henricii* | 1048.42±4.42 | 14 |  |  |  |
| AV7330  # | *P.chrysopogon* | 600.75±2.6 | 16 | U=0 | Pamela C. Rasmussen | Crocker Range, Sabah, Borneo |
|  | *P.monticola* | 795.09±5.62 | 17 |  |  |  |
| AV17210 | *P.chrysopogon* | 645.37±5.14 | 16 | U=240 | Julia Miller | Mulu NP, Sarawak, Borneo |
|  | *P.mystacophanos* | 738.42±5.02 | 15 |  |  |  |
| XC145661 | *P.chrysopogon* | 643.93±4.59 | 14 | U=0 | Yong Ding Li | Bentong, Pahang, Malaysia |
|  | *P.oorti* | 940.71±17.08 | 16 |  |  |  |
| AV13633 | *P.corvinus* | 608.19±5.96 | 17 | U=0 | Pamela C. Rasmussen | Gunung Halimun, Java |
|  | *P.armillaris* | 1306.37±15.73 | 18 |  |  |  |
| AV13635 | *P.corvinus* | 646.5±9.01 | 18 | U=324 | Pamela C. Rasmussen | Gunung Halimun, Java |
|  | *P.armillaris* | 1228.7±13.55 | 18 |  |  |  |
| XC93969 | *P.cyanotis* | 1670.47±17.39 | 16 | U=0 | Mike Nelson | Kaeng Krachan NP, Thailand |
|  | *P.asiaticus* | 839±10.37 | 16 |  |  |  |
| XC191831 | *P.duvaucelii* | 1938.15±6.75 | 8 | U=112 | Mike Nelson | Bukit Tinggi, Bentong, Pahang, Malaysia |
|  | *P.oorti* | 1024.28±18.52 | 14 |  |  |  |
| XC166572 | *P.duvaucelii* | 1747.68±22.22 | 8 | U=72 | Patrik Aberg | Danum Valley, Sabah, Borneo |
|  | *P.henricii* | 1070.62±11.16 | 9 |  |  |  |
| XC156582 | *P.eximius* | 1776.16±11.5 | 12 | F=788.92 | Marcus Braun | Poring, Sabah, Borneo |
|  | *P.duvaucelii* | 1903.83±35.93 | 11 |  |  |  |
|  | *P.mystacophanos* | 797.67±9.68 | 8 |  |  |  |
|  | *P.henricii* | 1017.97±19.65 | 10 |  |  |  |
|  | *P.chrysopogon* | 504.76±19.85 | 11 |  |  |  |
| XC94093 | *P.faiostrictus* | 867.61±11.32 | 16 | U=0 | Mike Nelson | Kaeng Krachan, Thailand |
|  | *P.cyanotis* | 1853.65±25.96 | 18 |  |  |  |
| XC104369 | *P.faiostrictus* | 870.59±8.71 | 16 | U=256 | David Farrow | Kaeng Krachan NP, Thailand |
|  | *P.cyanotis* | 1837.06±15.98 | 16 |  |  |  |
| XC165457 | *P.faiostrictus* | 770.15±15.35 | 15 | U=0 | Richard Dunn | Kaeng Krachan NP, Thailand |
|  | *P.cyanotis* | 1707.67±12.74 | 16 |  |  |  |
| XC200723 | *P.faiostrictus* | 886.07±10.3 | 15 | U=0 | Frank Lambert | Cat Tien NP, Vietnam |
|  | *P.cyanotis* | 1808.02±12.2 | 16 |  |  |  |
| XC41766 # | *P.faiostrictus* | 843.37±8.12 | 16 | U=224 | John Van der Woude | Khao Yai NP, Thailand |
|  | *P.incognitus* | 1163.09±8.52 | 14 |  |  |  |
| XC30040 | *P.henricii* | 1117.31±7.33 | 18 | U=4.95 | David Edwards | Danum Valley, Sabah, Borneo |
|  | *P.chrysopogon* | 613.46±4.47 | 16 |  |  |  |
| XC191839 | *P.henricii* | 1072.52±8.47 | 17 | U=319 | Mike Nelson | Bukit Tinggi, Bentong, Pahang, Malaysia |
|  | *P.oorti* | 965.82±12 | 19 |  |  |  |
| AV12434 | *P.lagrandieri* | 1304.57±25.64 | 7 | U=105 | Filip Verbelen | Cat Tien NP, Vietnam |
|  | *P.faiostrictus* | 821.71±7.51 | 15 |  |  |  |
| XC126899 | *P.lagrandieri* | 1331.26±4.18 | 17 | U=272 | Patrik Aberg | Cat Tien NP, Vietnam |
|  | *P.cyanotis* | 1819.05±15.33 | 16 |  |  |  |
| XC149873 | *P.lineatus* | 828.96±31.8 | 6 | U=13 | Niels Poul Dreyer | Nameri NP, Assam, India |
|  | *P.asiaticus* | 932.69±19.02 | 18 |  |  |  |
| XC125846 # | *P.lineatus* | 796.4±21.82 | 12 | U=24 | Sudipto Roy | Narendrapur, West Bengal, India |
|  | *P.asiaticus* | 948.8±24.98 | 16 |  |  |  |
| XC125498 | *P.malabaricus* | 822.26±4.57 | 15 | U=0 | Sudipto Roy | Molem NP, Goa, India |
|  | *P.viridis* | 1202.91±11.98 | 20 |  |  |  |
| XC44845 | *P.malabaricus* | 740.68±3.39 | 14 | U=0 | Sander Bot | Molem NP, Goa, India |
|  | *P.viridis* | 1213.95±14.65 | 15 |  |  |  |
| XC155689 | *P.monticola* | 813.46±4.24 | 20 | U=300 | Jason Anderson | Tambunan, Sabah, Borneo |
|  | *P.eximius* | 1655.48±9.87 | 15 |  |  |  |
| XC167254 | *P.monticola* | 734.63±2.67 | 18 | U=0 | Patrik Aberg | Mt. Kinabalu, Sabah, Borneo |
|  | *P.pulcherrimus* | 1468.4±18.67 | 22 |  |  |  |
| XC177802 | *P.mystacophanos* | 557.57±2.5 | 15 | F=754.82 | Johannes Fischer | Murung Raya, Central Kalimantan, Borneo |
|  | *P.duvaucelii* | 1536.99±27.43 | 15 |  |  |  |
|  | *P.henricii* | 1098.95±13.71 | 17 |  |  |  |
| XC196836 | *P.mystacophanos* | 734.03±10.4 | 15 | U=0 | Frank Lambert | Kubah NP, Sarawak, Borneo |
|  | *P.duvaucelii* | 1618.14±20.51 | 16 |  |  |  |
| XC64672 | *P.mystacophanos* | 848.22±5.88 | 17 | U=323 | Iain Woxvold | Barito River, Central Kalimantan, Borneo |
|  | *P.rafflesii* | 496.97±7.18 | 19 |  |  |  |
| XC196838 # | *P.rafflesii* | 462.64±1.92 | 16 | F=1327.24 | Frank Lambert | Kubah NP, Sarawak, Borneo |
|  | *P.mystacophanos* | 763.84±5.94 | 14 |  |  |  |
|  | *P.duvaucelii* | 1706.55±29.54 | 16 |  |  |  |
| XC21038 | *P.rafflesii* | 507.44±2.85 | 15 | F=1891.57 | Martjan Lammertink | Matan, West Kalimantan, Borneo |
|  | *P.mystacophanos* | 706.03±31.99 | 16 |  |  |  |
|  | *P.duvaucelii* | 2067.48±25.52 | 4 |  |  |  |
| XC196840 | *P.rafflesii* | 458.42±4.77 | 13 | F=2070.08 | Frank Lambert | Kubah NP, Sarawak, Borneo |
|  | *P.mystacophanos* | 806.72±18.97 | 8 |  |  |  |
|  | *P.duvaucelii* | 1953.71±22.33 | 16 |  |  |  |
| XC19626 | *P.rubricapillus* | 712.14±3 | 18 | U=324 | David Farrow | Udawattakele, Kandy, Sri Lanka |
|  | *P.flavifrons* | 1295.47±8.17 | 18 |  |  |  |
| XC154602 | *P.virens* | 1287.8±4.38 | 9 | U=135 | Hans Matheve | Doi Chiang Dao, Thailand |
|  | *P.asiaticus* | 975.83±17.25 | 15 |  |  |  |
| XC88665 | *P.virens* | 1462.04±4.32 | 9 | F=510.91 | Norbu | Shemgang, Bhutan |
|  | *P.franklinii* | 1039.51±8.01 | 8 |  |  |  |
|  | *P.asiaticus* | 958.63±17.17 | 10 |  |  |  |
| XC44888  # | *P.viridis* | 1178.56±23.91 | 17 | U=0 | Sander Bot | Indira Gandhi NP, Tamil Nadu, India |
|  | *P.malabaricus* | 690.65±1.7 | 14 |  |  |  |

**Supplementary Table S3:** Phylogenetic signal in morphological traits of Asian barbets. Note that all morphological traits are more divergent than expected under Brownian evolution (K<1).

| Trait | Blomberg’s K | P-value |
| --- | --- | --- |
| Beak length | 0.3508305 | <0.001 |
| Beak width | 0.3474846 | <0.001 |
| Beak depth | 0.3116673 | <0.001 |
| Tail length | 0.3273927 | <0.001 |
| Tarsus length | 0.6375699 | <0.001 |
| Body length | 0.6999196 | <0.001 |
| Wing chord length | 0.6639401 | <0.001 |
